# New Insights Into Diffuse Sclerosing Osteomyelitis: Is There a Role of ANA and Vitamin B6?

**DOI:** 10.1111/odi.70028

**Published:** 2025-08-04

**Authors:** Katharina Theresa Obermeier, Tim Hildebrandt, Ina Dewenter, Wenko Smolka, Eric Hesse, Philipp Poxleitner, Sven Otto

**Affiliations:** ^1^ Department Oral & Maxillofacial Surgery and Facial Plastic Surgery Ludwig Maximilians University Munich Germany; ^2^ Institute of Musculoskeletal Medicine University Hospital, LMU Munich Martinsried Germany

**Keywords:** ANA‐titer, autoimmunic disease, diffuse sclerosing osteomyelitis, jaw, osteomyelitis, vitamin B6

## Abstract

**Object:**

Diffuse sclerosing osteomyelitis is a poorly understood chronic disease, which appears predominantly in the mandible. Female patients are more often affected than men. DSO is an ultra‐rare disease and incidence is unknown; diagnosis can be very challenging; pathogenesis is poorly understood.

**Subjects and Methods:**

In this prospective study, blood samples were collected from a total of 25 patients and analyzed to detect altered laboratory values to have insight into possible pathomechanism. Patients were divided into three groups: Group A: patients with DSO, Group B: patients with osteomyelitis and medication‐related osteonecrosis, and Group C: healthy control group.

**Results:**

44% of all patients in group A had elevated Vitamin B6 levels. Compared to groups B and C, these levels were statistically significantly higher (*p* = 0.011, *p* = 0.015). 23 patients had increased (92%) antinuclear‐antibody‐titer (ANA‐Titer); in group B, 52% of all patients, and in the healthy control group, 44%.

**Conclusions:**

Elevated ANA titers and elevated vitamin B6 levels provide a possible insight into the pathomechanisms of DSO. Therefore, in the search for the pathogenesis and etiology of DSO, autoimmune processes should also be considered.

## 1 Introduction

Diffuse sclerosing osteomyelitis is a poorly understood chronic disease, which appears predominantly in the mandible. Usually, the corpus and ramus are affected and may include the condyle in some cases. The adjacent soft tissue can be affected by inflammation as well. Female patients are more often affected than males (Jacobsson 1979). Due to the fact that DSO is an ultra‐rare disease and its incidence is unknown, diagnosis can be very challenging (Müller‐Richter et al. 2009). DSO can occur isolated to the jawbone or as part of the SAPHO syndrome ( **s**ynovitis, **a**cne, **p**ustulosis, **h**yperostosis, and **o**steitis) (Kahn et al. 1994). Radiological findings include areas of bone sclerosis combined with periosteal reaction, a blurred nerve canal, and thickened cortical bone (van de Meent et al. 2018). Histopathological DSO is similar to fibrous dysplasia of the jaw and can be a challenge in diagnosis for pathologists (Jia et al. 2023).

Clinical symptoms present very heterogeneous. Patients report swelling of the face, which sometimes leads to an asymmetry. Trismus and severe pain are described. Patients describe heavy pain, which can increase up to 8–10 (VAS‐scale) on the visual analog scale (Otto et al. 2015). The occurrence of the disease in episodes is typical. Patients report symptom‐free intervals followed by attacks (Yoshii et al. 2001).

Normally, patients with the symptoms described above first receive radiological imaging (OPT, DVT and, if necessary, dental MRI). A skeletal scintigraphy is performed if there is a suspicion of saphoid syndrome. A bone biopsy is taken as standard to rule out other bone diseases. The diagnosis of DSO is therefore a combination of clinical symptoms, radiological diagnosis, nuclear medicine, and histopathological sample (van de Meent et al. 2018). Once the diagnosis has been made, patients can be treated with antiresorptive therapy (ibandronate or denosumab) (Otto et al. 2015). Considering the symptoms and the sterile inflammatory process, it is reasonable to assume that DSO may have an autoimmune genesis. Antinuclear antibody (ANA) titers are commonly used as markers in the diagnosis and monitoring of autoimmune diseases, reflecting underlying immune system activation (Zanussi et al. 2023). Over 90% of patients with systemic lupus erythematodes test positive for ANA titers (Li et al. 2022). In patients with Sjögren's disease, ANAtiters correlate with increased inflammation and systemic involvement (Shao et al. 2024). Emerging evidence also suggests a potential role for vitamin B6, an essential cofactor in immune function, in the modulation of autoimmune responses (Sande et al. 2019). Studies including patients with rheumatoid arthritis show that vitamin B6 status correlates with disease activity (Sande et al. 2019). It is also shown that levels of vitamin B6 change during therapy with TNF‐alpha inhibitors (Sande et al. 2019). Vitamin B6 levels and ANA titers are particularly interesting in patients with DSO due to their potential impact on immune function. ANA titers are commonly associated with autoimmune disorders, indicating a heightened immune response.

To our knowledge, there are no studies evaluating vitamin status and autoantibodies in DSO patients. The aim of the study was to investigate the relationship between vitamin B6 levels and ANA titers in patients with DSO to evaluate whether elevated ANA titers and increased vitamin B6 levels may serve as potential markers for the presence of DSO and to observe potential underlying mechanisms of this rare bone disease.

In this prospective study, blood samples were collected from a total of 25 patients and analyzed to detect characteristically altered laboratory values in patients with DSO.

## 2 Material and Methods

All patients who underwent treatment or check‐ups due to DSO in our department in 2023 and 2024 were included in the study. This prospective cohort study was approved by the institutional review board of the University Hospital of Munich, Germany (Munich, Germany; UE Nr 23‐0285). Informed consent was obtained from all subjects. Only patients with legal age were included in this study. In accordance with the statement of the ethics committee, the inclusion criteria were a diagnosis of DSO. DSO was diagnosed according to the following algorithm, which is used as standard in our department: clinical symptoms of patients (swelling, pain in episodes), radiological characteristics (blurred nerve canal, sclerotic bone and cortical bone thickness) as well as histopathological examination performed by two experts. Patients with other diseases of the jaw were excluded, as well as patients with severe liver or kidney disease. All patients underwent blood collection as part of the routine blood sample. Table 1 gives an overview of the blood values taken. The laboratory values of the Institute of Laboratory Medicine LMU, Munich, Germany, were selected as reference and are shown in Table 1. We included two control groups to compare the laboratory values, resulting in 3 groups included in this study. These are listed below:

- Group A: Patients with Diffuse Sclerosing Osteomyelitis.
- Group B: Patients with osteomyelitis of the jaw.
- Group C: Healthy control group.

**TABLE 1:** Labaratory values.

Laboratory value	Reference values	Average
Electrolytes		
Sodium	135–145 mmol/L	139.83 (± 1.95)
Potassium	3.5–5.0 mmol/L	4.63 (± 0.30)
Calcium	2.05–2.65 mmol/L	2.38 (± 0.08)
Anorganic phosphate	2.5–4.8 mg/dL	3.6 (± 0.44)
Magnesium	0.66–1.07 mmol/L	0.87 (± 0.06)
Iron	60–180 μg/dL	78.91 (± 36.51)
Liver values		
Gamma‐GT	< 39 U/L	32.17 (± 13)
ALT	< 34 U/L	21.08 (± 9.45)
AST	< 34 U/L	19.83 (± 5.24)
Hematology (blood count)		
Leucocytes	4.0–11.0G/l	7.54 (± 2.39)
Erythrocytes	3.96–5.16 T/L	4.69 (± 0.29)
Hemoglobin	11.5–15.4 g/dL	13.85 (± 1.18)
Hematocrit	0.346–0.453A L/L	0.41 (± 0.03)
MCV	80.0–95.5 fL	87.87 (± 3.67)
MCH	26.1–32.6 pg	29.52 (± 1.42)
MCHC	31.9–35.5 g/dL	34.33 (± 3.04)
Platelets	176–391 G/L	−259 (± 73.63)
Immunology		
ANA	< 1:100	
Antimitochondriale AB	< 1:100	
CCP (cycl. Citr. Peptid)	< 5:0 U/mL	
Ds‐DNS‐AK	< 100 IE/mL	
C3	0.9–1.8	
C4	0.1–0.4	
Transglutaminase‐AB	< 20 U/mL	
Endocrinology		
TSH	0.27–4.20 μU/mL	1.61 (± 0.64)
Vitamin		
Folic acid	4.4–31.0 ng/mL	
Vitamin B12 (Cobolamin)	197–771 pg/mL	
Vitamin A (Retinol)	30–80 μg/dL	
Vitamin B1 (Thiamin)	28.0–85.0 μg/mL	
Vitamin B2 (Riboflavin)	136–370 μg/L	
Vitamin B3 (Nicotin Acid)	8–52 μg/L	
Vitamin B6 (Pyridoxine)	8.7–27.2 μg/L	
Vitamin D3 (Cholecalciferol)	50–100nmol/l	
Vitamin E (Tocopherol)	5.0–20.0 mg/L	
Vitamin C (ascorbic acid)	4–15 mg/L	

Patient of Group C was matched to Group A considering age and gender. Only patients without any general diseases were included. All patients were treated due to tooth extraction or esthetic treatment in our department.

### 2.1 Statistical Analysis

The collected data was arranged using a Microsoft Excel spreadsheet (Microsoft Excel Version 16.0, Microsoft Corporation, Albuquerque, NM, USA).

Statistical analysis was conducted using SPSS 24 version 4.0 (SPSS Inc., Chicago, IL, USA). Thus, data shown in figures and tables are descriptive; mean values and standard deviation were calculated. To compare all groups statistically, the Mann–Whitney *U*‐test and the Chi‐Square test were performed. Statistical significance was defined as *p* < 0.05.

## 3 Results

### 3.1 Group A: Patients With Diffuse Sclerosing Osteomyelitis

#### 3.1.1 Demographic Data

Overall, 25 patients with DSO were included in this study. Nineteen patients were female (76%), 41.72 ± 17.2 years at first diagnosis, and six patients male (24%), 41.7 ± 25.1 years at first diagnosis. Total medium age of first diagnosis in all patients amounted 39.8 years ± 18.7. None of the patients reported a dietary supplementation. Average BMI amounted 25.6 kg/m^2^ (range from 18.3 to 27.8 kg/m^2^). Four patients (16%) suffered from hypothyroidism, which was treated with L‐thyroxine. In addition, six patients (24%) suffered from hypertension which was treated with ACE‐inhibitors. No patient suffered from diabetes and no metabolic diseases were reported. Seven of the patients (28%) reported a vegetarian diet. Overall, the patients had a balanced diet. All patients ate three meals a day. Ten patients (40%) presented with localisation of DSO found in the right mandible jaw and 15 patients (60%) on the left side: In three patients (12%) the condyle was affected. All patients reported episodes of pain and symptom‐free episodes. The average pain level in an acute episode of DSO was reported 6–8 on the VAS‐scale (Visual‐pain scale). Twelve patients had been already treated at least one time with antiresorptive therapy (ibandronic acid 6 mg). In five patients ibandronic acid has been given one time, in one patient two times, in five patients three times and in one patient eight times. In one patient denosumab has been applied instead of ibandronate every 6 months.

Table 1 gives an overview of all taken laboratory values by using mean values and standard deviation. None of the patients suffered from iron deficiency anemia; iron values and Hb‐levels were within the normal values.

#### 3.1.2 Inflammatory Marker

In six patients, blood samples were taken during episodes of pain. All other patients had their blood taken as part of normal routine diagnostics. Five patients showed increased CRP (c‐reactive protein) serum concentrations. The CRP levels showed no difference, regardless of whether the patient was experiencing an acute pain episode or presented with a symptom‐free episode (Table 2). In all patients, leucocyte levels were normal.

**TABLE 2:** Patients of group A with abnormal laboratory values.

Patient	Gender	Age	CRP	Vitamin B9 (folic acid)	Vitamin B 6 (pyridoxal)	Ds‐DNA‐antibody	C3 complement	ANA‐Titer	Present pain episode
Patient 1	Female	39.00	0.2	**4.1**	22.9	< 10	1.21 C3 **0.55 C4**	**1:400**	0
Patient 2	Female	45.00	0.3	7	19.9	< 10	1.15 C3 0.33 C4	**1:800**	0
Patient 3	Female	27.00	**1.3**	**3.3**	**49.1**	< 10	**1.82 C3** **0.45 C4**	**1:400**	0
Patient 4	Female	25.00	**1.2**	**3.2**	16.8	11	1.47 C3 0.3 C4	**1:100**	1
Patient 5	Female	58.00	**0.7**	**3.9**	16.4	< 10	1.31 C3 0.37 C4	**1:100**	0
Patient 6	Female	58.00	0.2	> 20	**138**	< 10	1.36 C3 0.11 C4	**1:100**	0
Patient 7	Male	16.00	0.1	**3.6**	24.8	29	1.65 C3 0.23 C4	**1:200**	0
Patient 8	Male	20.00	0.1	13.3	**32.3**	< 10	1.45 C3 0.11 C4	**1:200**	0
Patient 9	Female	35.00	0.1	**3.8**	18.6	**110**	1.08 C3 0.14 C4	**1:400**	1
Patient 10	Female	26.00	**2.1**	12.6	22.6	< 10	1.54 C3 0.13 C4	**1:100**	1
Patient 11	Male	81.00	**0.8**	10.3	17	< 10	1.23 C3 0.36 C4	**1:200**	0
Patient 12	Female	27.00	< 0.5	6.7	**39.6**	< 10	1.15 C3 0.15 C4	**1:200**	1
Patient 13	Female	40	0.1	10.8	**35.6**	< 10	1.15 C3 0.17 C4	1:100	0
Patient 14	Male	62	0.8	2.4	15.7	< 10	1.59 C3 0.3 C4	1:200	0
Patient 15	Female	61	0.1	16.7	**51.2**	15	1.16 C3 0.22 C4	1:200	0
Patient 16	Male	31	0.1	13.4	**30.7**	< 10	1.15 C3 0.26 C4	1:100	0
Patient 17	Female	59	**2.5**	5.4	18.8	< 10	1.58 C3 0.24 C4	1:100	1
Patient 18	Female	19	0.1	18.5	22.5	< 10	1.64 C3 **0.55 C4**	1:100	1
Patient 19	Female	30	0.1	**3.7**	**40.5**	< 10	1.05 C3 **0.45 C4**	1:100	1
Patient 20	Male	24	**0.5**	7.5	23.6	< 10	1.33 C3 0.35 C4	1:200	0
Patient 21	Female	74	0.1	76.3	**76.3**	< 10	1 C3 0.23 C4	1:100	0
Patient 22	Female	28	0.1	17.5	13	< 10	1.07 C3 0.24 C4	1:100	0
Patient 23	Female	30	0.1	6.8	20.3	< 10	0.86 C3 0.16 C4	1:200	0
Patient 24	Female	70	0.1	5.3	**30.7**	< 10	1.08 C3 0.32 C4	—	0
Patient 25	Female	58	0.1	5.7	**39**	< 10	1.27 C3 0.28 C4	—	0

#### 3.1.3 Vitamin Levels

**FIGURE 1:**
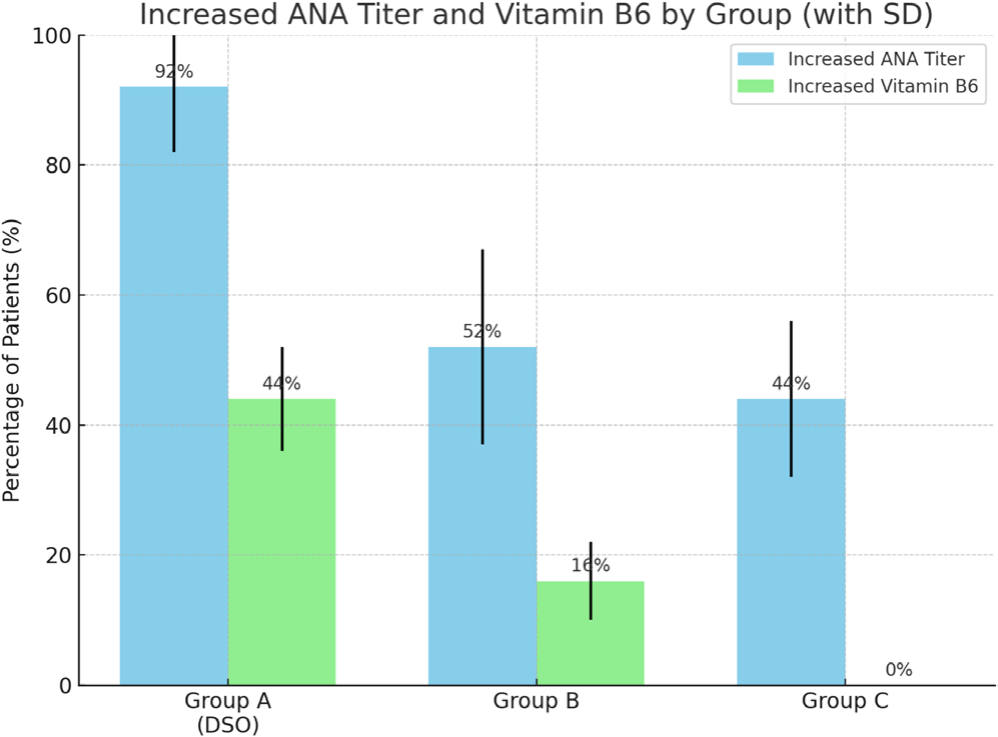
Overview of increased ANA titer and Vitamin B6 levels.

Two patients (8.7%) showed decreased vitamin D levels. Vitamin B12 was elevated in two patients (8.7%) and vitamin C level was reduced in 3 patients (13%). Folic acid was reduced in 8 patients (34.8%). Vitamin B6 (pyridoxal) was increased in 11 patients (44%). Figure 1 shows Vitamin B6 levels considering Groups 1, 2, and 3.

#### 3.1.4 Rheumatology Laboratory

CCP (anti‐CCP‐antibodies) showed no anomalies as well as ENA Screen, Histon antibodies, AMA (IFT) and anticytoplasmase.

Ds‐DNA‐antibody was increased in one patient (4%). C3 and C4 complement factor was increased in four patients (16%).

ANA‐Titer were increased in 23 patients (92%) included in this study.

### 3.2 Group B: Patients With Osteomyelitis of the Jaw and MRONJ

Overall, 25 patients were included in Group B. Eighteen patients were female (72%) and seven patients (28%) were male. Nine patients (36%) suffered from bacterial osteomyelitis of the jaw and 16 (64%) from MRONJ (Medication‐related osteonecrosis of the jaw). The medium age at the time of inclusion in the study was 60.28 ± 18.8 years. Patients with osteomyelitis were treated with antibiotic medication (sultamicillin and clavulan acid). Patients with MRONJ were treated surgically with modeling osteotomy, sequesterotomy, or partial resection and primary wound closure as well as antibiotic therapy. Twelve patients (48%) reported a vitamin D supplementation. Average BMI amounted to 23.3 kg/m^2^ (range from 19.1 to 24.8 kg/m^2^). Four patients (16%) suffered from diabetes, and no metabolic diseases were reported. Five of the patients (20%) reported a vegetarian diet. Overall, the patients had a balanced diet. All patients ate three meals a day. Blood samples were taken prior to surgery. Vitamin E, B1, B2, B3, B12, and C showed normal values.

#### 3.2.1 Inflammatory Marker

Twelve patients (48%) showed increased CRP (c‐reactive protein) levels (Table 3). In all patients, leucocyte levels were normal.

**TABLE 3:** Group B patients with osteomyelitis of the jaw and MRONJ.

Patient	Gender	Age	CRP	Vitamin B9 (folic acid)	Vitamin B6 (pyridoxal)	Ds‐DNA‐antibody	C3 complement	ANA‐Titer
Patient 1	Female	84	0.39	7.4	19.8	< 10	1.16 C3 0.25 C4	—
Patient 2	Female	47	0.1	**2.9**	103	< 10	1.06 C3 0.4 C4	**1:100**
Patient 3	Male	56	**0.6**	10.9	27.6	< 10	1.43 C3 0.47 C4	—
Patient 4	Female	74	**1.3**	**4.7**	24.9	< 10	1.13 C3 0.24 C4	—
Patient 5	Female	75	**0.9**	6.5	**36.8**	< 10	1.45 C3 0.39 C4	—
Patient 6	Male	48	**0.5**	11.7	**35.4**	< 10	0.89 C3 0.37 C4	**1:100**
Patient 7	Male	55	**0.9**	**2**	23.5	< 10	1.13 C3 0.38 C4	—
Patient 8	Male	84	< 0.1	**< 2.0**	25.3	< 10	0.94 C3 0.17 C4	**1:100**
Patient 9	Female	73	**3.4**	**2.7**	13.6	< 10	1.48 C3 0.35 C4	—
Patient 10	Female	90	**1.7**	**4.3**	25.1	< 10	C3 0.17 C4	**1:200**
Patient 11	Male	47	0.1	8.7	22.8	< 10	1.28 C3 0.37 C4	—
Patient 12	Male	55	**0.8**	**3.7**	13.5	< 10	1.07 C3 0.43 C4	**1:200**
Patient 13	Female	56	0.2	5.6	16.9	< 10	1.11 C3 0.17 C4	—
Patient 14	Female	75	**0.9**	7.9	14.9	< 10	1.29 C3 0.23 C4	**1:200**
Patient 15	Male	63	0.2	16	24	< 10	1.29 C3 0.46 C4	**1:100**
Patient 16	Female	62	0.2	10.9	26.1	< 10	1.36 C3 0.19 C4	**1:200**
Patient 17	Female	75	0.4	**2.8**	15.8	< 10	1.45 C3 0.2 C4	—
Patient 18	Female	59	1	**2.1**	13.8	< 10	1.2 C3 0.31 C4	**1:100**
Patient 19	Female	44	**8.8**	**2.12**	13.8	< 10	1.3 C3 0.21 C4	**1:100**
Patient 20	Female	33	0.2	7.6	19.4	< 10	1.26 C3 0.16 C4	—
Patient 21	Female	20	0.1	**3.8**	23.6	< 10	0.98 C3 0.18 C4	**1:400**
Patient 22	Female	24	0.2	**3**	**95.6**	< 10	1.17 C3 0.25 C4	**1:100**
Patient 23	Female	58	**0.6**	> 20.0	**209**	< 10	1 C3 0.15 C4	—
Patient 24	Female	60	0.5	**4.5**	19.5	< 10	1.24 C3 0.36 C4	—
Patient 25	Male	90	**8.3**	**4.4**	24.1	< 10	1.15 C3 0.14 C4	**1:100**

#### 3.2.2 Vitamin Levels

One patient (4%) showed decreased vitamin D levels. Folic acid was reduced in 14 patients (56%). Vitamin B6 (pyridoxal) was increased in four patients (16%).

#### 3.2.3 Rheumatology Laboratory

CCP (anti‐CCP‐antibodies) showed no anomalies as well as ENA Screen, histoneantibodies, AMA (IFT) and anticytoplasmase.

Ds‐DNA‐antibody was increased in one patient. C3 and C4 complement factor was increased in no patient.

ANA‐Titer were increased in 13 patients (52%) included in this study.

### 3.3 Group C: Healthy Control‐Group

Overall, 25 patients were included in Group C. 14 (56%) were female and 11 (44%). All patients in the control group were healthy and treated in the department due to tooth extraction. Median age at the time of inclusion in the study was 51.3 ± 19.2 years.

Four patients (16%) reported a vitamin D supplementation. Average BMI amounted to 24.6 kg/m^2^ (range from 19.8 to 25.6 kg/m^2^). No patient suffered from diabetes, and no metabolic diseases were reported. Eight of the patients (32%) reported a vegetarian diet, and One patient reported a vegan diet (4%). Overall, the patients had a balanced diet. All patients ate three meals a day. Vitamin E, B1, B2, B3, B12, and C showed normal values.

#### 3.3.1 Inflammatory Marker

Four patients (16%) showed increased CRP levels (Table 4). In all patients, leukocyte levels were normal.

**TABLE 4:** Group C (Healthy Control Group).

Patient	Gender	Age	CRP	Vitamin B9 (folic acid)	Vitamin B6 (pyridoxal)	Ds‐DNA‐antibody	C3 complement	ANA‐Titer
Patient 1	Female	62	< 0.1	9.8	18.9	< 10	1.15 C3 0.4 C4	—
Patient 2	Female	52	0.2	5.8	22.5	< 10	1.16 C3 0.37 C4	—
Patient 3	Male	50	< 0.1	**< 2.0**	20.7	< 10	1.15 C3 0.21 C4	**1:100**
Patient 4	Female	26	< 0.1	> 20	22.8	< 10	1.19 C3 0.11 C4	**1:100**
Patient 5	Male	55	2	15.5	6.8	< 10	1.13 C3 0.38 C4	—
Patient 6	Male	68	0.5	20	11.4	< 10	1.07 C3 0.3 C4	—
Patient 7	Male	48	0.1	7.6	18.1	< 10	1.31 C3 0.19 C4	—
Patient 8	Female	22	0.1	**< 2.0**	19.9	< 10	0.93 C3 0.35 C4	**1:200**
Patient 9	Male	35	0.8	6.8	35.8	< 10	0.87 C3 0.27 C4	—
Patient 10	Male	39	0.1	**< 2.0**	22.6	< 10	1.54 C3 0.27 C4	—
Patient 11	Male	92	0.8	4.8	22.2	< 10	1.12 C3 0.25 C4	**1:100**
Patient 12	Male	22	0.8	5.9	37.2	< 10	0.84 C3 0.31 C4	—
Patient 13	Female	54	5.5	**4.8**	21.1	< 10	1.1 C3 0.32 C4	**1:200**
Patient 14	Female	67	1.10	5.6	21.6	< 10	1.32 C3 0.34 C4	—
Patient 15	Male	55	3.20	5.40	29.9	< 10	1 C3 0.16 C4	**1:400**
Patient 16	Female	70	0.20	13	26.5	< 10	1.16 C3 0.22 C4	—
Patient 17	Female	62	0.60	5.6	20.3	< 10	1.1 C3 0.28 C4	—
Patient 18	Female	20	0.20	**4.6**	38.3	< 10	1.14 C3 0.21 C4	—
Patient 19	Female	20	0.40	10	36.6	< 10	1.59 C3 0.31 C4	**1:100**
Patient 20	Female	67	0.10	9.2	31.7	< 10	1.05 C3 0.19 C4	**1:200**
Patient 21	Male	75	1.20	17.8	25.3	< 10	1.52 C3 0.25 C4	—
Patient 22	Male	52	0.20	5.8	24.3	< 10	1.27 C3 0.24 C4	**1:100**
Patient 23	Female	61	0.20	**4**	42.1	< 10	1.54 C3 0.38 C4	**1:100**
Patient 24	Female	69	0.10	7.5	22.8	< 10	1.06 C3 0.27 C4	—
Patient 25	Female	58	0.28	> 20	20	< 10	1 C3 0.15 C4	**1:100**

#### 3.3.2 Vitamin Levels

One patient (4%) showed decreased vitamin D levels. Folic acid was reduced in 6 patients (24%). Vitamin B6 (pyridoxal) was normal in all patients (100%).

#### 3.3.3 Rheumatology Laboratory

CCP (anti‐CCP‐antibodies) showed no anomalies as well as ENA‐Screen, histone‐antibodies, AMA (IFT) and anticytoplasmase.

Ds‐DNA‐antibody was increased in one patient. C3 and C4 complement factor was increased in no patient.

ANA‐Titer were increased in 11 patients (44%) included in this study.

### 3.4 Statistical Analysis

Table 5: Statistical analysis of Groups A, B, and C; Figure 1 shows Groups A, B, and C considering ANA titer and Vitamin B6 levels.

**TABLE 5:** Statistical analysis of Groups A, B, and C.

Group A versus B	
CRP	*p* < 0.01
Vitamin B9	*p* = 0.35
Vitamin B6	*p* = 0.011
ANA‐Titer	*p* = 0.004
Group A versus C	
CRP	*p* = 0.13
Vitamin B9	*p* = 0.55
Vitamin B6	*p* = 0.015
ANA‐Titer	*p* = 0.001

#### 3.4.1 Power Analysis

Additionally, we performed a power analysis considering ANA‐titer and vitamin B6 level. Effect sizes were estimated using Cohen's w for chi‐square tests, yielding values of approximately 0.47 and 0.52, respectively, which show large effects. The statistical power for both outcomes exceeds 90%, indicating that the study is adequately powered to detect the observed group differences. Additional analysis for vitamin B6 using a Mann–Whitney *U*‐test (approximated via *t*‐test) confirmed that a large effect size (*r* = 0.5) also achieves over 90% power with the current sample size.

#### 3.4.2 Bonferroni‐Correction

We added Bonferroni correction to pairwise comparison, resulting in an adjusted significance threshold of α = 0.05/3 ≈ 0.0167, meaning *p*‐values (Vitamin B6: Group A vs. Group B *p* = 0.011 and Group A vs. Group C *p* = 0.015, ANA‐Titer: Group A vs. Group B *p* = 0.004, Group A vs. Group C *p* = 0.001) are below. Therefore, the differences in Vitamin B6 levels and ANA‐titer between the groups are still considered significant after correction.

## 4 Discussion

DSO is a rare disease of the jaw bone. Etiology and pathogenesis are still unclear, and due to the rarity of the disease prospective studies are a challenge for researchers. This prospective clinical cohort study includes 25 patients with the primary diagnosis of DSO treated in the department of craniomaxillofacial surgery, LMU, Munich, Germany. The average age of patients at the time of primary diagnosis amounted to 39.8 ± 18.7 years, which is similar to other studies (Shao et al. 2024). The gender ratio man: woman was 19:6, which is also described as typical for this clinical syndrome in the literature (Sande et al. 2019; Matharu et al. 2020).

Multiple etiological theories for DSO have been proposed. It has been hypothesized that DSO is a low‐grade infection caused by Propionibacteria and afterwards turned into a chronic sterile infection (Muraoka et al. 2023). Malstrom et al. made the hypothesis that DSO is a hyperactive immunological response due to the fact that they found increased IgM antibodies in blood samples (Berglund et al. 2015). Also, chronic Musculus masseter overuse resulting in chronic tendoperiostitis and afterwards in DSO was postulated (Jacobsson et al. 1982). However, none of the theories definitively reveal the ultimate etiology and pathogenesis of DSO. This study aimed to examine laboratory values in patients with DSO and shows abnormalities in them. There was a statistically significant difference between Group A and Group B considering CRP serum concentration; Group B had higher CRP levels (*p* < 0.01).

Increased CRP level could be caused by many different factors: bacterial inflammation, viral diseases, heart attacks or apoplex or reactive after surgeries (Jacobsson et al. 1982; Pearson et al. 2003). None of the patients had any of the factors mentioned above. Low CRP level can be an expression of chronic inflammation (Desborough 2000). Since DSO is a chronic inflammatory disease, the elevated CRP levels could be an expression of this. Low increased CRP levels might also be an expression of stress related symptoms and due to the high pain levels in our patients be a possible reason (Desborough 2000).

Two patients showed decreased Vitamin D levels, which are interpreted as chronic Vitamin D deficiency caused by too little UV radiation (Del Giudice and Gangestad 2018) and due to higher age than average age in our study. In general, folic acid (vitamin B9) is known to play an important role during pregnancy and in neural tube defects (Bikle 2014).

In our study, 44% of all patients in group A had elevated Vitamin B6 levels. Compared to groups B and C, these levels were statistically significantly higher (*p* = 0.011, *p* = 0.015). Increased Vitamin B6 levels can be initiated by higher supplementation of Vitamin B6 in nutrition. However, all patients reported normal and balanced nutrition.

Vitamin B6 (Pyridoxal) plays an important role in several metabolism circles and is a co‐factor in over 100 enzymatic reactions, including carbohydrate metabolism, amino acid metabolism, particularly homocysteine, gluconeogenesis, glycogenolysis, and lipid metabolism. Vitamin B6 is also involved in the critical functioning of cells. It plays a significant role in transamination, decarboxylation, and the initial steps of porphyrin synthesis (Valentin et al. 2018; Meier et al. 1981).

In autoimmune diseases, increased vitamin B6 levels are described. Nemazannikova et al. (2018) stated that increased vitamin B6 levels could be typical in patients with multiple sclerosis (Brown et al. 2022). Plasma PLP is also involved in the risk of cardiovascular disease and some cancers and is inversely associated with numerous inflammatory markers (Nemazannikova et al. 2018). Abnormal vitamin B6 levels, measured by low circulating concentrations of the metabolically active form pyridoxal 5′‐phosphate (PLP), are well‐known in rheumatoid arthritis (RA) (Ueland et al. 2017). Circulating PLP has been found to be inversely correlated with C‐reactive protein (CRP). Autoimmune diseases, such as rheumatoid arthritis, have increased catabolism of B6, resulting in higher demand for dietary supplementation of B6 (Chiang et al. 2003). Nevertheless, vitamin B6 levels can be influenced by dietary intake, liver function, and metabolic status, and thus have an influence on the data collected. In our study, in one patient, CRP and vitamin B6 levels were increased at the same time; in two patients, low CRP levels and increased vitamin B6 levels were found at the same time; and in four patients, low vitamin B6 levels and elevated CRP levels were found at the same time. Overall, it can be suspected that vitamin B6 seems to play an immunomodulatory role in the pathogenesis of DSO, which requires further clinical studies.

In 23 patients (92%) the antinuclear‐antibody‐titer (ANA‐Titer) was increased. ANAs are a type of antibodies that bind to cellular components in the nucleus (DNA, RNA, and nucleic acid‐protein complexes) (Sande et al. 2019). ANAs are classified into two groups: the anti‐DS‐DNA‐antibody (antibody to histone and DNA) and the other group targets nuclear antigens (Nemazannikova et al. 2018). ANAs are one of a few specific markers in systemic autoimmune diseases. The presence of ANA increases the risk for an autoimmune disorder and autoimmune connective tissue disorder (Kumar et al. 2009). On the other hand, 33% and more of the healthy population present with increased ANA levels and without any systemic autoimmune disorder (Xiao et al. 2022). ANA positivity can be detected in up to 20%–30% of healthy individuals, particularly at low titers, and can also be seen in infections, malignancies, and even in response to certain medications (Kumar et al. 2025). ANA titers are increased in 27.7% of patients with present malignancies (Solans‐Laqué et al. 2004). In breast cancer and liver cancer, increased ANA titers are described (Tan and Zhang 2008). Hsu et al. (2023) showed that ANA status can predict immune‐related events in patients with liver cancer undergoing PD‐1 therapy. They showed that the increased ANA titers shortened the time between PD‐1 therapy and immune‐related adverse effects (Hsu et al. 2023).

Several medications can increase the level of ANA titer as well. ANA elevation can be found in patients with procainamide, hydralazine and minocycline (Tan and Zhang 2008). In our study no patient took these medications. ANA plays also an important role in infections. Increased titer can be found in patients with hepatitis, Epstein‐bar Virus. (Yee et al. 2004). Just as immune complex formation during infections can increase ANA titer, particularly in chronic diseases (Horvatits et al. 2020). In our study 44% of all patients in the healthy control‐group (Group C) showed positive ANA, which is quite similar to the literature as well as in Group B with 52% positive ANA. This is slightly higher than reported in literature, but could be a result of inflammatory processes going on in diseases like osteomyelitis and necrosis of the jaw. All positive results must be interpreted with the existing clinical manifestations to establish a diagnosis. In our patients 92% had increased ANA‐titer and one patient had increased anti‐DS‐DNA‐titer and existing clinical manifestation that may be a hint that DSO could be an autoimmune disease. In all patients, disease appeared in episodes of pain followed by symptom‐free intervals. In addition, in four patients also elevated C3 and C4 complement factor has been found, which is also increased in autoimmune diseases such as lupus erythematosus and autoimmune hepatitis (Kumar et al. 2025; Tebo 2017).

Although the cohort of included patients is small due to the rareness of the disease, there is a hint that inflammatory and immunomodulatory processes play an important role. Although the rarity of the disease often results in small sample sizes and reduced statistical power, this study may give possible insights into the pathomechanism. Overall, there is a hypothesis that DSO may be linked to autoimmune diseases. However, this association remains unproven and requires further investigation. This study should serve as a pilot study and provide the impetus for further projects in this field. Therefore, in the search for the pathogenesis and etiology of DSO, autoimmune processes should also be considered and investigated in further studies.

## 5 Conclusion

Elevated ANA titers, slightly elevated CRP levels, and elevated vitamin B6 levels provide a possible insight into the pathomechanisms of DSO. This data could be a hint that DSO might be caused by autoimmune processes. Additionally, ANA and vitamin B6 should not be considered a specific biomarkers for DSO; rather, they should assist in finding the diagnosis and excluding differential diagnoses. There is a need for future cohort studies with larger patient populations. These studies are essential for validating the current findings, which could support, in the future, personalized therapeutic approaches.

## Author Contributions

**Katharina Theresa Obermeier:** conceptualization, investigation, writing – original draft, methodology, visualization, writing – review and editing, software, formal analysis. **Tim Hildebrandt:** conceptualization, investigation. **Ina Dewenter:** investigation, formal analysis. **Wenko Smolka:** conceptualization, formal analysis, writing – review and editing. **Eric Hesse:** methodology, validation, writing – original draft, supervision. **Philipp Poxleitner:** validation, methodology. **Sven Otto:** conceptualization, investigation, formal analysis, supervision.

## Conflicts of Interest

The authors declare no conflicts of interest.

## Data Availability

The datasets generated and/or analyzed during the current study are not publicly available due to the anonymization process, but are available from the corresponding author on reasonable request.
